# Impact of conservation tillage on wheat performance and its microbiome

**DOI:** 10.3389/fpls.2023.1211758

**Published:** 2023-08-21

**Authors:** Ida Romano, Natacha Bodenhausen, Gottlieb Basch, Miguel Soares, Hanna Faist, Friederike Trognitz, Angela Sessitsch, Marcé Doubell, Stéphane Declerck, Sarah Symanczik

**Affiliations:** ^1^ Department of Agricultural Sciences, Division of Microbiology, University of Naples Federico II, Naples, Italy; ^2^ Department of Soil Sciences, Research Institute of Organic Agriculture (FiBL), Frick, Switzerland; ^3^ MED – Mediterranean Institute for Agriculture, Environment and Development, University of Évora, Évora, Portugal; ^4^ AIT Austrian Institute of Technology, Tulln, Austria; ^5^ Mycology, Earth and Life Institute, Université Catholique de Louvain, Louvain-la-Neuve, Belgium

**Keywords:** tillage, wheat genotype, amplicon sequencing, soil microbiome, irrigation, fertilization

## Abstract

Winter wheat is an important cereal consumed worldwide. However, current management practices involving chemical fertilizers, irrigation, and intensive tillage may have negative impacts on the environment. Conservation agriculture is often presented as a sustainable alternative to maintain wheat production, favoring the beneficial microbiome. Here, we evaluated the impact of different water regimes (rainfed and irrigated), fertilization levels (half and full fertilization), and tillage practices (occasional tillage and no-tillage) on wheat performance, microbial activity, and rhizosphere- and root-associated microbial communities of four winter wheat genotypes (Antequera, Allez-y, Apache, and Cellule) grown in a field experiment. Wheat performance (i.e., yield, plant nitrogen concentrations, and total nitrogen uptake) was mainly affected by irrigation, fertilization, and genotype, whereas microbial activity (i.e., protease and alkaline phosphatase activities) was affected by irrigation. Amplicon sequencing data revealed that habitat (rhizosphere vs. root) was the main factor shaping microbial communities and confirmed that the selection of endophytic microbial communities takes place thanks to specific plant–microbiome interactions. Among the experimental factors applied, the interaction of irrigation and tillage influenced rhizosphere- and root-associated microbiomes. The findings presented in this work make it possible to link agricultural practices to microbial communities, paving the way for better monitoring of these microorganisms in the context of agroecosystem sustainability.

## 1 Introduction

Wheat is one of the major crops worldwide, and its production is estimated to increase by 11% by 2026 (Cangioli et al., 2022). Current management practices mainly rely on the application of external inputs such as pesticides for pest and disease control, mineral fertilizers to improve plant nutrition and biomass, and often irrigation to avoid water stress conditions. In combination with intensive soil tillage, these management practices may significantly reduce microbial diversity (Tsiafouli et al., 2015), whose key functions for crop production are widely recognized.

Soil is a non-renewable natural resource. Its health is the result of biotic and abiotic processes and is linked to several interactions (Frąc et al., 2018). These interactions have a significant impact on microbial activity, which supports essential soil processes (Frąc et al., 2018). Microorganisms are the most abundant and diverse group among all organisms living in soils (Gagelidze et al., 2018), playing crucial roles in maintaining ecological functions such as the decomposition of organic matter, energy flow, and nutrient cycling (Prakash et al., 2020). However, microbial communities are highly susceptible to soil changes, such as disturbances due to tillage, irrigation, and fertilization (Li et al., 2021), i.e., practices which are considered essential to achieve profitable crop yields.

Long-term fertilization has been shown to alter soil pH, available phosphorus (P), total carbon (C), and nitrogen (N), resulting in significant variations in soil microbiomes (Yuan et al., 2016). Furthermore, N availability largely impacts soil microbial communities (Ramirez et al., 2010; Liu et al., 2021). Li et al. (2021) have shown that N fertilization may directly or indirectly change the soil microbiome by decreasing bacterial diversity and shifting toward a more active and copiotrophic microbial community. On the other hand, irrigation can alter the composition and activity of soil microbial communities through changes in soil water content, which leads to changes in the movement and availability of soil nutrients (Li et al., 2021). In the case of the wheat–maize rotation field trial performed by Yang et al. (2018), irrigation had a higher impact on the structure of the soil microbial community than N fertilization. Specifically, irrigation increased the abundance, diversity, and functionality of the bacterial community, whereas fertilization had minor effects.

In addition to fertilization and irrigation, tillage practices have also been shown to impact the soil microbiome. Applying the principles of Conservation Agriculture (CA) can benefit soil microorganisms by enhancing soil organic matter contents, which help in maintaining soil moisture and buffering against temperature peaks due to permanent soil cover by crop residues (Mathew et al., 2012; Li et al., 2022c; Lv et al., 2023). By contrast, intensive tillage practices create strong disturbances in soil biological, chemical, and physical properties, including bulk density, water holding capacity, pore size distribution, and aggregation, creating different habitats for soil microorganisms resulting in shifts of the soil microbial community structure (Mathew et al., 2012; Li et al., 2020; Srour et al., 2020). Kraut-Cohen et al. (2020) have shown that soil tillage significantly changed the relative abundance of soil microorganisms, including a reduction of heterotrophic bacteria possibly involved in decomposing complex organic matter and an increase in aerobic taxa. In addition to changes in composition, they observed an increase in hydrolytic and redox microbial activities in no-till soils at two independent field sites.

The understanding of the complex and dynamic interactions between soil habitats such as root (endosphere) and rhizosphere (root surrounding area) and their associated microbiota has significantly improved over the past few decades, notably through molecular ecology approaches, which have considerably expanded the scientific knowledge on soil microbial communities, showing that these two ecological habitats exhibit distinct phylogenetic structures, indicating different microbial populations (Philippot et al., 2013; Fitzpatrick et al., 2018; Kuzyakov and Razavi, 2019; Liu et al., 2019). Microbiome management is a highly promising tool for the sustainable intensification of agricultural systems (Bakker et al., 2012; French et al., 2021; Jing et al., 2022; Yang et al., 2023) and the development of biodiversity-based agriculture (Duru et al., 2015; Struik and Kuyper, 2017; Tittonell et al., 2020). However, harnessing the rhizosphere microbiome through plant breeding (Peiffer et al., 2013; Kusstatscher et al., 2021) and agroecosystem management is still in its infancy. Plant traits can lead to differences in the composition and functioning of the rhizospheric and endophytic microbiome, and certain plant genotypes promote beneficial microbiomes, supporting the hypothesis that there are genotype-specific plant–microbe interactions (Peiffer et al., 2013; Philippot et al., 2013; Ulbrich et al., 2021; Escudero-Martinez and Bulgarelli, 2023). Therefore, when selecting a specific crop genotype, it is important to consider the association with beneficial microorganisms.

Today, most studies have focused on the individual effect of single management practices on soil microbial communities, whereas only few studies investigated the combined effects of N fertilization, irrigation, and tillage (Li et al., 2021; Cangioli et al., 2022). Rillig et al. (2019) also emphasized the importance of studying the interplay of multiple factors to tackle future challenges of soil management. The present study aims to increase our understanding on how the composition and functioning of the root and rhizosphere microbiome can change in response to agricultural management practices and how this, in turn, impacts wheat growth. By combining different agroecological approaches, the goal is to promote biological processes in agroecosystems to maximize the delivery of key ecosystem services (Duru et al., 2015). We hypothesized that the combination of sustainable agricultural management practices affects the composition of the soil microbiome, thereby enhancing microbiome functioning and hence plant growth. To challenge this hypothesis, we investigated the activity of enzymes involved in P and N cycling, as well as the composition of bacterial and fungal communities using amplicon sequencing of the rhizosphere and root samples collected from four winter wheat varieties grown under different fertilization, irrigation, and tillage practices.

## 2 Materials and methods

### 2.1 Field experiment

Four winter wheat varieties (i.e., *Triticum aestivum* var. Antequera, Allez-y, Apache, and Cellule) were grown under two fertilization levels: full fertilized (recommended N rate) and half fertilized (recommended N rate/2), two water regimes (rainfed and irrigated), and two tillage treatments: occasional tillage (tilled) and no-tillage (no-till). Based on different potential grain yield expectations under rainfed (3 t/ha) and irrigated conditions (6 t/ha), the recommended N rate was calculated as follows: 30 kg N/t expected grain yield minus the mineral N content (kg N min/ha) determined before the first top dressing, which was 30 kg N/ha. Therefore, the full fertilization rate was 60 and 150 kg N/ha under rainfed and irrigated conditions, respectively. Natural rainfall during the vegetation period between 16/11/2018 and 18/06/2019 was 209 mm, complemented, under irrigation, with four irrigation events of 7 mm each, three during April and one at the beginning of May.

The trial was organized in a split-plot design with fertilization and genotype nested in tillage and water regime as main factors and three replicated plots each (dimension: 4.5 × 8 m) (**Supplementary Figure S1**). The experiment was conducted in Beja, Alentejo, Portugal (37.954396, −7.830063) on a clay loam soil (clay 32.9%; silt 29.0%; sand 38.1%) under a dry Mediterranean climate (average annual precipitation of approx. 555 mm). The soil was characterized by 1.191% soil organic C, 0.072% organic N, low available P (Olson method), and medium available potassium (K, 1 M NH_4_OAc method) concentrations in mg/g dry soil of 0.0198 and 0.1205 at 0–20 cm, 0.013 and 0.0938 at 20–40 cm, and 0.0088 and 0.0678 at 40–60 cm, respectively. The tilled treatment consisted of one pass of a tine cultivator at 20–25-cm depth and two passes of a disc harrow to prepare the final seedbed. Plots were supplied with a PK fertilizer at a rate of 80 kg of P_2_O_5_/ha and 80 kg of K_2_O/ha prior to sowing. Sowing took place in 12/12/2018. A rate of 200 kg of seed/ha was considered corresponding to an approximate rate of 440 seeds/m^2^ in order to achieve a minimum of 350 plants/m^2^. All plots were seeded with a John Deere 750 No Till Drill (single disc opener) with a 17-cm row spacing. A postemergence herbicide was applied once (Atlantis [0.6% (p/p) iodosulfuron-methyl sodium, 3% (p/p) mesosulfuron-methyl, 9% (p/p) mefenpyr-diethyl]), in a concentration of 420 g/ha diluted in 200 l of water, 44 days after sowing on 25/01/2019. Nitrogen fertilization was applied as ammonium nitrate in two top dressings on 08/02/2019 and 27/03/2019, respectively, 58 and 105 days after sowing. The rate was split equally between the two top dressings, 30 kg N/ha per top dressing under rainfed conditions and 75 kg N/ha per top dressing under irrigation. The trial was established on a no-till field (no-till for 3 years) with barley as preceding crop. The barley straw was removed, and only the stubble with a height of 10–15 cm remained on the field before the trial was established.

### 2.2 Sample collection

At the early grain filling stage (principal growth stage 7. BBCH), 21/05/2019, rhizospheric soil, roots, and aboveground wheat biomass were collected. Three to four wheat plants were excavated from a depth of 0–10 cm using a spade keeping a 1.5-m distance to the respective plot margin. For enzyme analysis, rhizosphere soil was collected by shaking off loosely attached soil from the excavated root system. For molecular analyses, roots with attached rhizosphere soil were transferred into 50-ml tubes and kept on ice until further processing in the laboratory. For N content analysis of spikes and leaves plus stalks, a 50 × 51-cm area in each plot encompassing three planted rows and located away from the plot margins was cut with a cordless shrub shear. Samples were taken at growth stage BBCH 69 (i.e., flowering complete, some dehydrated anthers may remain) in different dates for the four varieties: Antequera (26/04/2019), Cellule (30/04/2019), and Apache and Allez-y (08/05/2019). The final harvest of the plot was carried out on 18/06/2019 with a small plot combine harvester. A grain sample for each plot was taken for N content analysis.

### 2.3 Soil and plant-related analyses

Spikes were separated from leaves and stalks and counted. Harvested biomass was oven dried at 70°C for 48 h. Once dried, the two plant components were weighed and finely grinded with a rotor mill. A subsample was then taken and analyzed for N content using a Leco FP-528 Nitrogen/Protein Determinator following the Dumas method. Grain N content analysis followed the same protocol as above.

The rhizosphere soil for enzyme analyses was sieved at 2 mm. One subsample was used directly to assess the dry matter (DM) content of the soil by drying at 105°C to constant weight. The remaining rhizosphere soil was stored at −20°C before measuring the potential activity of (i) proteases using a spectrophotometric stop rate assay adapted from Schinner et al. (1991) and Ladd and Butler (1972) and (ii) acid phosphomonoesterases using an adapted spectrophotometric assay of Margesin (1993) and Tabatabai and Bremner (1969) as described in **Supplementary Data**.

The 50-ml tubes containing the roots with the attached rhizosphere soil were shaken in 25 ml of sterile water for 3 min. Roots were removed and stored for further processing; the remaining soil–water suspension was centrifuged at 4,000×g for 10 min. After decanting the supernatant, the sedimented rhizosphere soil was homogenized, lyophilized, and stored in a desiccator until DNA extraction. Root samples were washed with tap water, surface sterilized by submersion for 5 min in 2.5% NaOCl enriched with one drop of Tween 20, washed three times in sterile water, and dried at 85°C for 3 h. Cut roots were frozen at −80°C, homogenized twice for 1.5 min in a TissueLyser at 30 Hz in two different orientations, and stored at −20°C until DNA extraction.

### 2.4 DNA extraction, quantification of bacterial and fungal marker genes, amplicon sequencing, and data processing

DNA was extracted from 250 mg of rhizosphere soil or 50 mg of root powder with the DNeasy PowerSoil HTP 96 Kit (Qiagen, Hilden, Germany) following the supplier’s instructions and including negative controls (DNA extraction blanks). Total DNA concentration was measured using the Qubit fluorometric assay (Thermo Fisher Scientific, Waltham, USA), and extracts were stored at −20°C until further analyses.

Amplicon libraries were prepared in a two-step polymerase chain reaction (PCR) approach. The first PCR with Nextera-tagged primers (Illumina, Inc., USA) targeting bacterial communities (Faist et al., 2023) and CS1/CS2-tagged primers (Fluidigm, San Francisco, CA, USA) targeting the entire fungal community (fungi 1) was performed in technical triplicates using different DNA template concentrations (1:5, 1:10, 1:15 dilutions) to minimize the stochastic PCR effects of individual reactions using a SYBR Green approach (KAPA SYBR FAST qPCR Master Mix (2×) Universal Kit; Kapa Biosystems, Wilmington, MA, USA) on a CFX96 Touch Real-Time PCR Detection System (Bio-Rad Laboratories, Hercules, CA, USA). Primer sequences and PCR cycling conditions are given in **Supplementary Table 1**. Triplicated PCRs were pooled before further processing. Bacterial amplicons of 480 bp were separated from root DNA via gel electrophoreses and enriched via gel extraction. Fungal 1 amplicons were purified using a magnetic bead solution (https://openwetware.org/wiki/SPRI_bead_mix) and visualized on agarose gel (1.5%). The second PCR, library preparation, and paired-end sequencing on an Illumina MiSeq sequencing platform (Illumina, San Diego, CA, USA) were performed at the Genome Quebec Innovation Center (Montreal, Canada) for fungal 1 amplicons and at the AIT for bacterial amplicons according to the amplicon guidelines provided by Illumina. For the second fungal amplicon library (fungi 2), primers supposed to be specific for Glomeromycota were used. The fungal 2 amplicon library was prepared in a three-step PCR approach, also including three technical replicates per sample. For the first PCR, the primer pair SSUmAf and LSUmAr described by Krüger et al. (2009) were used. PCR products were directly used as templates for a nested PCR using the modified forward primer LSUD2mod (Senés-Guerrero et al., 2020) and the LSUmBr reverse primer mix (Krüger et al., 2009) with attached Nextera overhang adapters for Illumina MiSeq. Amplicons were visualized on a 2% agarose gel before pooling and purified using AMPure XP beads (Qiagen, Hilden, Germany). In the third PCR, each sample was indexed with primers of the Nextera XT Index Kit (Illumina, Inc, USA). The third PCR, library preparation, and paired-end sequencing on an Illumina MiSeq platform of fungal 2 amplicons were performed by the Next Generation Sequencing Facility at Vienna BioCenter Core Facilities (VBCF), member of the Vienna BioCenter (VBC), Austria. The raw sequencing data were uploaded to the Sequence Read Archive (SRA) from NCBI (https://www.ncbi.nlm.nih.gov/sra/docs) under the project number PRJNA985128 (http://www.ncbi.nlm.nih.gov/bioproject/985128).

The bioinformatics analysis was conducted at the Scientific Computer Cluster Euler at ETH Zurich. USEARCH v11.0.667 (Edgar, 2010) was used to remove phiX and merge read pairs with a minimum overlap of 30 bp and a minimum merge length of 100 bp. Primer sequences were removed, and paired reads were size-selected, quality-filtered, and denoised using USEARCH v11.0.667 (Edgar, 2010). Removal of chimaera and clustering into zero radius operational taxonomic units (ZOTUs) were done via UNOISE (Edgar, 2016a). Additionally, clustering at 97% sequence identity was done by UPARSE (Edgar, 2013). Taxonomy was assigned via SINTAX (Edgar, 2016b) using SILVA v128 (Quast et al., 2013) and UNITE_v82 (Bengtsson-Palme et al., 2013) as reference for the bacterial and fungal datasets at 0.85 and 0.5 tax filter identity thresholds, respectively.

### 2.5 Statistical analysis

The R statistical environment (R version 4.1.2) was used for data analysis using RStudio (Kronthaler and Zöllner, 2021). One-way ANOVA (p ≤ 0.05) was used to assess the difference in agronomic data and enzyme assay analyzing the factors wheat genotype, fertilization, water regime, and tillage. Microbial community data were organized and analyzed with R package phyloseq (McMurdie and Holmes, 2013) and vegan 2.5-6 (Oksanen et al., 2019). The quality of sequencing was controlled with rarefaction analysis using the rarecurve function from vegan package (**Supplementary Figure 2**). Alpha diversity was assessed with Shannon diversity. The Shannon–Weaver index (H) is calculated as follows: H = -sum pi * ln pi, where pi is the proportional abundance of species i. Additionally, from the Shannon–Weaver index, the diversity was calculated as follows: D  =  exp(H) (Jost, 2007; Bodenhausen et al., 2013). Beta diversity was examined by permutational multivariate analysis of variance (PERMANOVA) using the adonis function from vegan. Principal coordinate analysis (PCoA) on Bray–Curtis dissimilarities was used to visualize the differences between samples. For visualization of microbial community structure, unconstrained ordination by principal component analyses (PCAs) based on clr-transformed ZOTU tables was performed, followed by distance-based redundancy analyses (db-RDA) constraining for statistically significant factors identified in PERMANOVA and conditioning for block. The abundant community was defined as the OTUs that were present in 99% of the samples for bacteria, in 97% for fungi 1, and 65% for fungi 2; percentages were defined based on prevalence plots. Filtering was performed with the metagMisc (version 0.5.0) R package with the parameter fprev.trh set according to prevalence plot observations. Differential abundance testing was performed with the DESeq2 (version 1.34.0) R package with parameter fitType=“parametric” (Love et al., 2014). Prior to analysis, OTUs were grouped at the genus level, then the identified taxa were filtered using a combination of false discovery rate (FDR) and log fold change (logFC) and applying with two sets of thresholds (FRD < 0.001 and logFC > 2), to identify differentially abundant genera between habitats. Heatmaps were generated in R using the package ggplot2. The metabolic function was predicted by Tax4Fun analysis through the Kyoto Encyclopedia of Genes and Genomes (KEGG) database (Wang et al., 2019; Gugliucci et al., 2023). Analysis focused on the differences of predicted abundances of protease and phosphate genes in rhizosphere samples. ANOVA (p ≤ 0.05) was used to assess the difference in predicted abundance taking into account the factor tillage, water regime, fertilization and genotype. Heatmaps were generated in R using the package pheatmap 1.0.12 (Kolde, 2019).

## 3 Results

### 3.1 Agronomic performance of winter wheat

The effect of agricultural management practices on winter wheat grain yield and N partitioning in different plant parts (spikes, leaves and stalk, grains) is presented in **Table 1** and **Supplementary Table 2**. Wheat yield was significantly affected by genotype, fertilization level, and water regime and was higher in Cellule compared with Allez-y, Apache, and Antequera; higher under full fertilization than under half fertilization; and higher under irrigated compared with rainfed conditions. Leaf and stalk N concentrations were significantly affected by fertilization and genotype and was higher under full compared with half fertilization and higher in Allez-y, Apache, and Cellule compared with Antequera. The spike N concentration was significantly influenced by genotype, fertilization level, and water regime and was higher in Allez-y and Apache compared with Antequera and Cellule, higher under full fertilization than under half fertilization, and higher under rainfed compared with irrigated conditions. Grain N concentration was significantly affected by genotype, fertilization, and water regime with similar patterns for the factor fertilization (full > half) and water regime (rainfed > irrigated) but with higher grain N concentrations in Antequera and Allez-y compared with Apache and Cellule. N uptake expressed in kg N per ha was only affected by fertilization with higher N uptake under full compared with half fertilization.

**Table 1:** Effect of agricultural management practices on yield and nitrogen (N) concentrations in spikes, leaves and stalk, grains, and total N uptake of four winter wheat genotypes grown under different water and fertilization regimes and tillage treatments.

	Yield (t/ha)	Spikes N (%)	Leaf and stalk N (%)	Grain N (%)	N uptake (kg N/ha)
Water regime					
Rainfed	1.99 ± 0.68	1.95 ± 0.22	1.27 ± 0.42	2.27 ± 0.33	91.5 ± 26.2
Irrigated	2.90 ± 0.63	1.88 ± 0.26	1.34 ± 0.28	2.00 ± 0.55	89.3 ± 43.2
F_ANOVA_	82.52 ***	7.98 **	2.87 ns	34.06 ***	0.17 ns
Tillage					
No-till	2.40 ± 0.81	1.92 ± 0.24	1.91 ± 0.25	2.11 ± 0.45	91.0 ± 40.2
Tilled	2.45 ± 0.81	1.31 ± 0.37	1.30 ± 0.35	2.17 ± 0.49	89.8 ± 30.6
F_ANOVA_	0.20 ns	0.42 ns	0.03 ns	1.51 ns	0.06 ns
Genotype					
Antequera	2.25 ± 0.59	1.66 ± 0.15	1.18 ± 0.32	2.29 ± 0.18	88.5 ± 29.2
Allez-y	2.13 ± 0.60	2.14 ± 0.16	1.42 ± 0.41	2.66 ± 0.19	85.6 ± 41.2
Apache	2.18 ± 0.72	2.01 ± 0.20	1.29 ± 0.36	1.93 ± 0.18	86.8 ± 41.5
Cellule	3.17 ± 0.86	1.85 ± 0.12	1.33 ± 0.37	1.68 ± 0.33	100.7 ± 38.8
F_ANOVA_	20.70 ***	71.74 ***	4.67 **	27.44 ***	1.71 ns
Fertilization					
Half-fertilized	2.40 ± 0.81	1.81 ± 0.22	1.04 ± 0.24	2.01 ± 0.55	66.3 ± 24.9
Full-fertilized	2.45 ± 81	2.02 ± 0.21	1.58 ± 0.24	2.27 ± 0.33	114.5 ± 27.4
F_ANOVA_	5.65 *	79.85 ***	137.28 ***	222.99 ***	81.96 ***

Values represent means ± standard error of three replicates. ns, not significant; *p < 0.05; **0.001 ≤ p < 0.01; ***p < 0.001.

### 3.2 Microbial activity

Microbial activity assessed by protease, alkaline, and acidic phosphatase activity is presented in **Table 2**. Protease activity was significantly affected by water regimes and higher under irrigated compared with rainfed conditions. Water regimes also affected alkaline phosphatase with higher activity in rainfed compared with irrigated conditions, whereas acidic phosphatase was not affected. No significant effects of tillage, genotype and fertilization regime were noticed on these factors.

**Table 2:** Protease, alkaline, and acidic phosphatase activity in the rhizosphere of four winter wheat genotypes grown under different water and fertilization regimes and tillage treatments.

	Protease (µg tyrosine/g dw soil)	Alkaline phosphatase (µg nitrophenol/g dw soil)	Acidic phosphatase (µg nitrophenol/g dw soil)
Water regime			
Rainfed	1360.8 ± 549.9	694.7 ± 124.1	235.9 ± 84.5
Irrigated	1647.8 ± 532.1	484.6 ± 140.2	253.1 ± 84.5
F_ANOVA_	6.74*	61.15 ***	1.41 ns
Tillage			
No-till	1481.0 ± 613.7	587.7 ± 162.4	243.1 ± 82.6
Tilled	1527.5 ± 499.8	591.6 ± 176.6	245.9 ± 57.5
F_ANOVA_	0.67 ns	0.02 ns	0.04 ns
Genotype			
Antequera	1412.8 ± 524.5	556.4 ± 164.3	224.6 ± 64.6
Allez-y	1412.8 ± 595.6	644.0 ± 162.0	251.9 ± 69.8
Apache	1614.2 ± 457.6	585.7 ± 175.1	242.7 ± 63.5
Cellule	1513.5 ± 648.8	572.5 ± 171.0	258.7 ± 83.2
F_ANOVA_	0.58 ns	0.12 ns	0.38 ns
Fertilization			
Half-fertilized	1410.3 ± 510.6	586.7 ± 156.3	249.2 ± 67.4
Full-fertilized	1598.2 ± 590.6	592.6 ± 182.1	239.8 ± 74.4
F_ANOVA_	2.89 ns	0.05 ns	0.52 ns

Values represent means ± standard error of three replicates. dw, dry weight; ns, not significant; *p < 0.05; ***p < 0.001.

### 3.3 Composition of microbial communities

**Figure 1:**
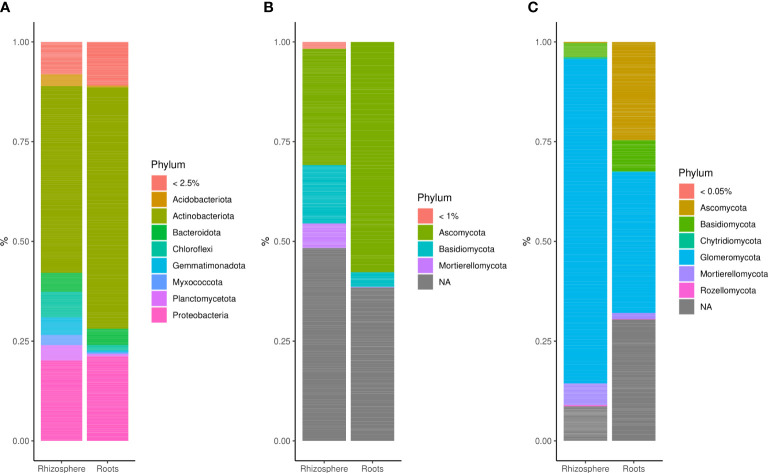
Bar plots showing the relative abundance of **(A)** bacterial phyla (>2.5%), **(B)** fungal 1 phyla (>1%), and **(C)** fungal 2 phyla (>0.05%) in the rhizosphere and root of winter wheat.

Amplicon sequencing yielded a total of 5,473,023 bacterial 16S, 5,441,306 fungal ITS (fungi 1), and 5,322,161 fungal LSU (fungi 2) reads obtained from 96 root and 96 rhizosphere samples. Sequences were annotated to 5,474 bacterial, 1,040 fungal 1, and 615 fungal 2 OTUs. Bacterial communities were dominated by the phylum *Actinobacteriota* (~25% up to 50%) followed by *Acidobacteriota*, *Bacteroidota*, *Chloroflexi*, *Gemmatimonadota*, *Myxococcota*, *Planctomycetota*, and *Proteobacteria* (**Figure 1A**). Fungal 1 communities were dominated by the phyla *Ascomycota* (from ~25% to 55%), *Basidiomycota* (<15%), and *Mortierellomycota* (<10%), and the percentage of unassigned sequences ranged between ~40% and 48% (**Figure 1B**). Rhizosphere-associated fungal 2 communities were dominated by *Glomeromycota* (~80%), whereas *Glomeromycota* (~35%), *Ascomycota* (~25%), and *Basidiomycota* (<10%) were most abundant in roots (**Figure 1C**). There was also a small representation of *Mortierellomycota* (from 2% to 5%), *Chytridiomycota* (<1%), and *Rozellomycota* (<1%).

### 3.4 Microbial diversity

**Figure 2:**
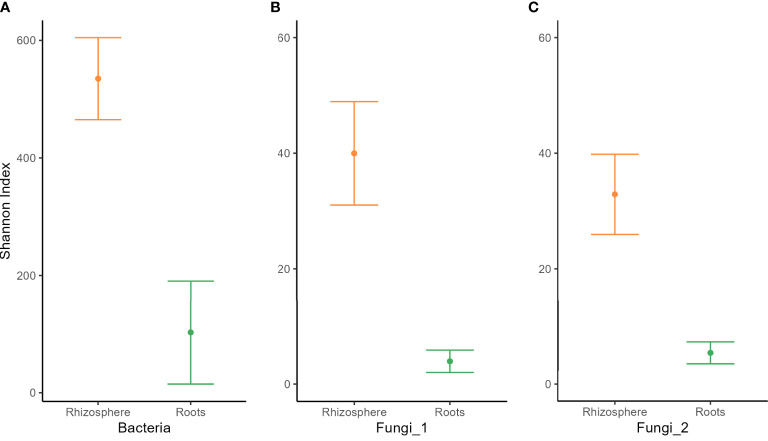
Alpha diversity of **(A)** bacterial,**(B)** fungal 1, and **(C)** fungal 2 communities in the rhizosphere (orange) and roots (green) of winter wheat. Means ±standard error of three replicates are shown.

**Figure 3:**
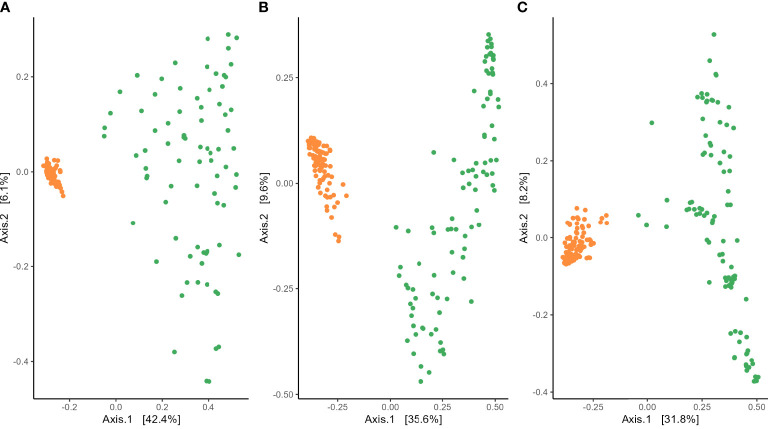
Beta diversity assessed with Bray–Curtis distance of **(A)** bacterial, **(B)** fungal 1, and **(C)** fungal 2 communities in the rhizosphere (orange) and roots (green) of winter wheat.

**Figure 4:**
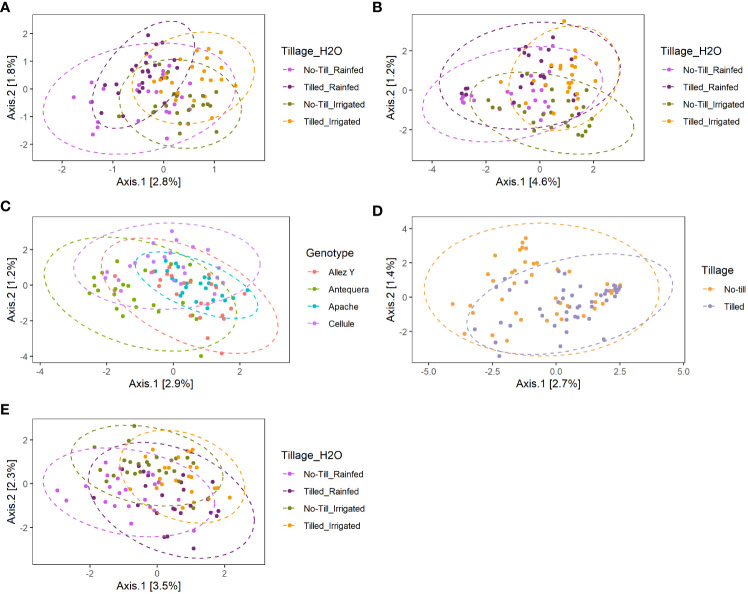
Constrained distance-based redundancy analysis (db-RDA) of bacterial **(A, B)**, fungal 1 **(C, D)** and fungal 2 **(E)** community composition in the rhizosphere (A, C, E) and roots **(B, D)** of winter wheat based on significant factors identified in PERMANOVA.

Analysis of variance of the Shannon index performed on habitat, wheat genotype, fertilization, water regime, and tillage revealed that microbial diversity was only affected by habitat (**Supplementary Table 3**). A significantly higher Shannon index was found in the rhizosphere compared with roots for bacterial and fungal communities (**Figure 2**). PERMANOVA analysis on beta-diversity performed on all experimental factors allowed to identify distinct bacterial and fungal community structures in the rhizosphere compared with roots (**Figure 3**; **Supplementary Table 4**). In addition, both habitats were analyzed separately to further evaluate the effect of the agricultural management on beta diversity (**Supplementary Table 4**). Constrained (db-RDA) ordinations of each individual habitat showed an apparent clustering of bacterial communities for both tillage practice and water regime (p < 0.001) (**Figures 4A, B**). In contrast, fungal communities showed habitat-specific clustering. Rhizosphere-associated fungal 1 communities were affected by genotype (p < 0.01), whereas root-associated ones were affected by tillage practice (p < 0.05) (**Figures 4C, D**). Fungal 2 communities associated with the rhizosphere were affected by tillage practice and water regimes (p < 0.01) (**Figure 4E**).

### 3.5 Differentially abundant and prevalent OTUs

**Figure 5:**
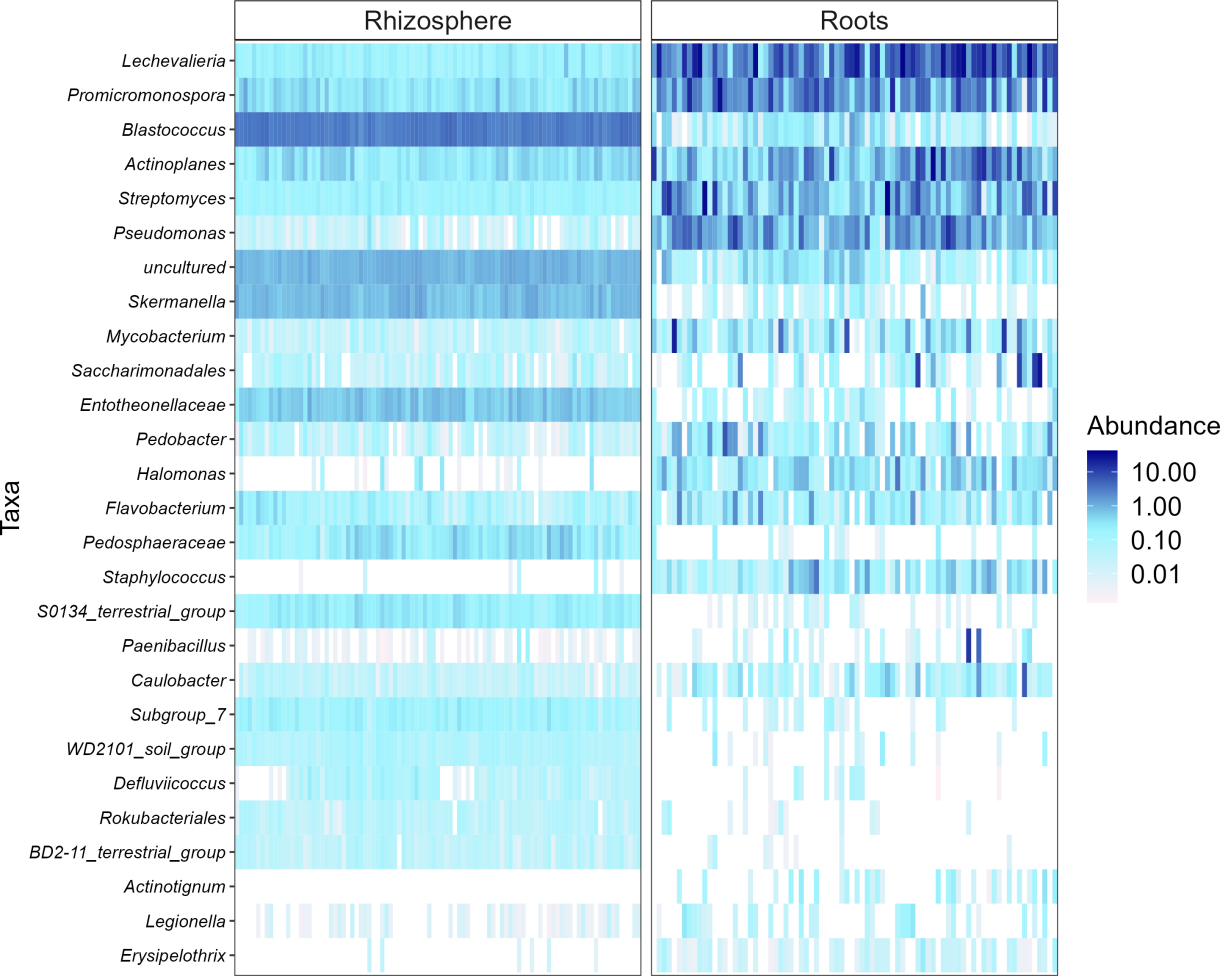
Heat plot showing the most abundant rhizosphere- and root-associated bacterial genera of winter wheat evaluated on combination of false discovery rate (FDR) and log fold change (logFC) using two sets of thresholds (FRD < 0.001 and logFC > 2).

**Figure 6:**
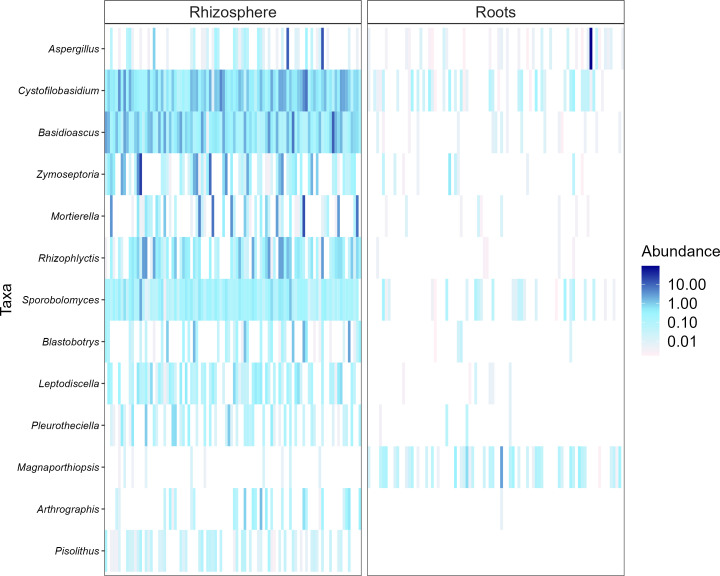
Heat plot showing the most abundant rhizosphere- and root-associated fungal 1 genera of winter wheat evaluated on combination of false discovery rate (FDR) and log fold change (logFC) using two sets of thresholds (FRD < 0.001 and logFC > 2).

**Figure 7:**
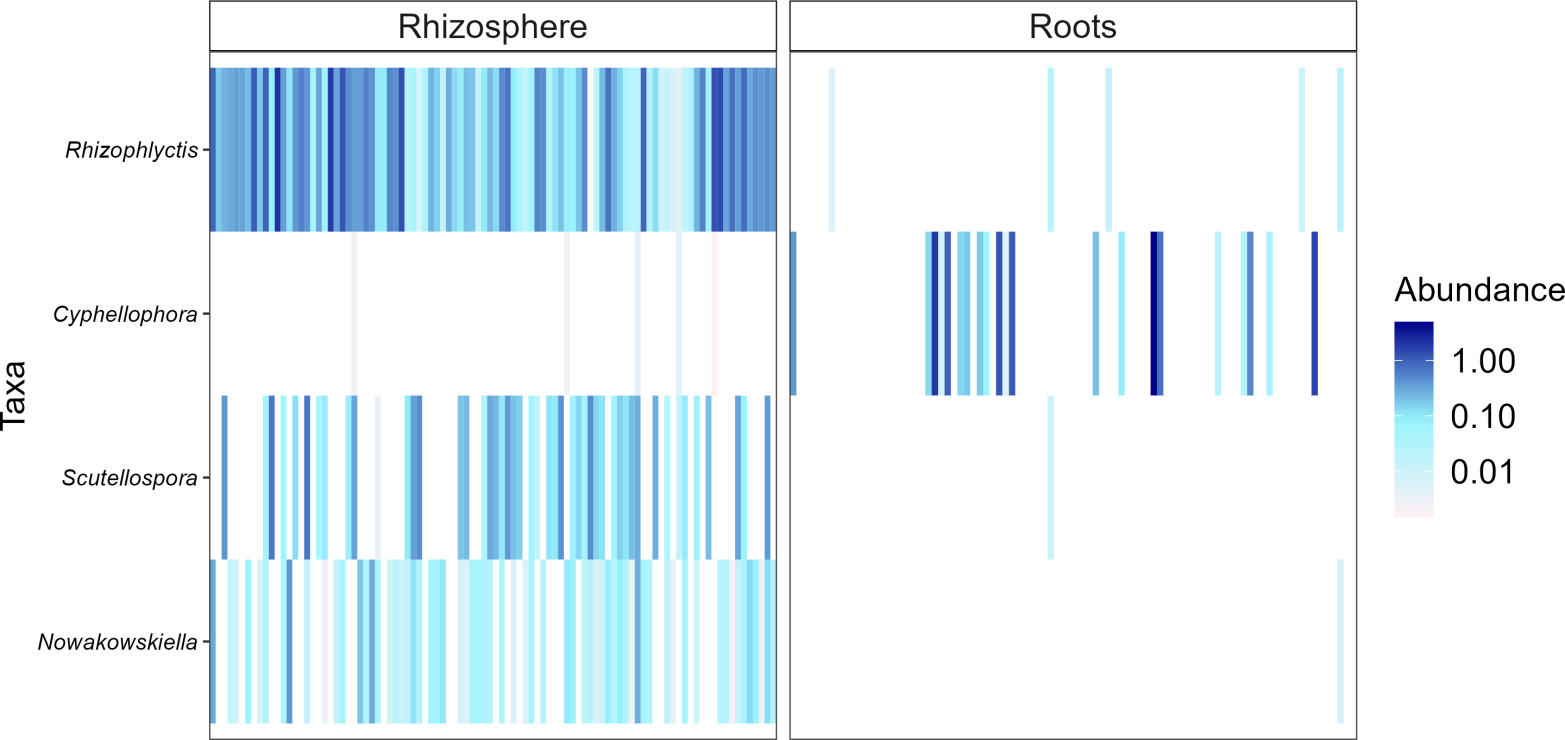
Heat plot showing the most abundant rhizosphere- and root-associated fungal 2 genera of winter wheat evaluated on combination of false discovery rate (FDR) and log fold change (logFC) using two sets of thresholds (FRD < 0.001 and logFC > 2).

Heatmaps, evaluated as a combination of false discovery rate (FDR) and log fold change (logFC) using two sets of thresholds (see “Materials and methods”), provided a good overview of differentially abundant bacterial and fungal genera (**Figures 5**–**7**). The generated heatmaps clustered the microbial communities based on their habitat as there were no effects of agricultural practices (tillage, water regime, fertilization) and plant genotype. This resulted in habitat-specific patterns of characteristic bacterial and fungal genera. Most abundant bacterial genera in the rhizosphere were *Blastococcus*, *Skermanella*, and *Entotheonellacea* and in roots *Lechevalieria*, *Promicromonospora*, *Actinoplanes*, *Streptomyces*, and *Pseudomonas* (**Figure 5**). Bacteria of the genera *Halomonas*, *Staphylococcus*, *Actinotignum*, and *Erysipelothrix* were specific for roots and lacked in rhizosphere, whereas *Pedosphaeraceae*, *Defluviicoccus*, and *Rokubacteriales* were characteristic for the rhizosphere. For fungal 1 communities, the genera *Cystofilobasidium*, *Basidioascus*, and *Sporobolomyces* were specific for the rhizosphere, whereas only *Magnaporthiopsis* was specific in roots (**Figure 6**). For fungal 2 communities, the genera *Rhizophlyctis*, *Scutellospora*, and *Nowakowskiella* were specific for the rhizosphere and only *Cyphellophora* was specific for roots (**Figure 7**). In addition to differentially abundant OTUs, also prevalent OTUs present in most of the samples and thus representing the core community were evaluated individually for roots and the rhizosphere (**Supplementary Figures 3**, **5**). The bacterial core community in roots present in 100% of the samples were composed of eight OTUs belonging to the genera *Lechevalieria*, *Promicromonospora*, *Actinoplanes*, *Streptomyces*, *Lechevalieria*, *Pseudomonas*, and *Bacillus*. The bacterial core community in the rhizosphere present in 100% of the samples were composed of 406 OTUs. The fungal 1 core community in roots present in 95% of the samples were composed of five OTUs belonging to the genera of which only one was assigned at the phylum level as *Ascomycota*. The fungal 1 core community in the rhizosphere present in 100% of the samples were composed of 13 OTUs belonging to Tremellomycetes, *Mortierellomycetes*, *Ascomycota*, *Geminibasidiomycetes*, and *Altenaria* and 7 unassigned fungi. The fungal 2 core community in roots present in 65% of the samples were composed of six OTUs belonging to *Pleosporales*, *Sordariomycetes*, *Xylariales*, and *Ascomycota* and one unassigned fungus. The fungal 2 core community in the rhizosphere present in 100% of the samples was composed of 20 OTUs belonging to *Glomeromycota* (7 OTUs), *Tremellomycetes* (3 OTUs), *Basidiomycota*, and 6 unassigned fungi.

### 3.6 Functional prediction analysis

We predicted functional profiles of proteases and phosphatases based on 16S rDNA gene sequencing data, and then we assessed differences between agricultural management practices in the rhizosphere of winter wheat (**Supplementary Figure 4**). ANOVA analyses identified a set of 14 predicted genes whose abundances were significantly affected by the experimental factors used in the study (**Supplementary Table 6**). In particular, predicted genes encoding ATP-dependent protease Clp (K03544), the cell division protease FtsH (K03798), a hypothetical protease (K14742), the isocitrate dehydrogenase phosphatase kinase (K00906), two pyrophosphatases (K01520 and K02428), the fructose 1–6 bisphosphate aldolase phosphatase (K01622), and the D-glycero-D-manno-heptose-1,7-bisphosphate phosphatase (K03273) were significantly affected by tillage practice (p < 0.05; **Supplementary Table 6**). On the other hand, the predicted genes encoding histidinol phosphatase (K01089), phytase (K01093), and diacylglycerol pyrophosphatase (K01521) were significantly influenced by fertilization (p < 0.05; **Supplementary Table 6**). Furthermore, the subtilase-type serine protease (K12685), the carboxyl terminal processing protease (K03797), and the hydrogenase 1 maturation protease (K03605) were found to be significantly affected by both tillage and fertilization (p < 0.05; **Supplementary Table 6**). Functional profiles of the bacterial communities were clustered into two major groups clearly associated with tillage with a higher abundance of predicted genes in the no-till system (**Supplementary Figure 4**).

## 4 Discussion

### 4.1 Effect of agricultural management on winter wheat yield and N uptake

The application of two fertilization regimes uniformly impacted wheat performance resulting in higher yields and N concentrations under full compared with half fertilization. This corroborates the study of López-Bellido and López-Bellido (2001), who also observed higher yields and N uptake in wheat grown under full N fertilization compared with half fertilization. These results are further consistent with the meta-analysis of Hossard et al. (2016) which reported higher wheat yields in conventionally wheat cropping systems compared with low-input and organically fertilized systems of Europe and North America. In contrast, water regime differentially affected yield and N concentration of spikes and grains with higher yield under irrigation and higher N concentrations under rainfed conditions. Considering that total N uptake was not affected by water regimes, the difference in N concentration observed in spikes and grains must be due to a dilution effect due to higher wheat biomass in irrigated plots, as already reported by Jarrell and Beverly (1981). Deficit irrigation is a strategy for crop production allowing the reduction of water use in agriculture. Nevertheless, it was not extensively applied, mainly due to the risk of yield loss (Yu et al., 2020). The meta-analysis of Li et al. (2022a) helped to quantify the impacts of deficit irrigation on yield of winter wheat and maize cultivated in northern areas of China. They observed that deficit irrigation leads to variable crop losses, which depend on crop type, hydrological conditions, and growing regions (Li et al., 2022a). In contrast to fertilization and irrigation, tillage practices did not affect yield and N uptake of winter wheat. Also, Pittelkow et al. (2015) who performed a meta-analysis on the effect of no-till on yields of various crops, observed the smallest negative impact of no-till on wheat. Similarly, López-Bellido and López-Bellido (2001) observed similar yield and N uptake of wheat grown under no-till and conventional tillage under Mediterranean conditions. Also Ruisi et al. (2014) found on average similar yields of durum wheat under no-till compared with conventional tillage comparing the results of field trials conducted over a period of 20 years. However, in contrast to our results, Ruisi et al. (2014) and Ruisi et al. (2016) observed a decrease in N uptake under no-till compared with conventional tillage due to a lower N availability in the soil. One potential reason for the lack of effects of tillage on wheat yield and N uptake might be that the trial was newly established on a no-till field site. Thus, tillage practices were implemented only a few months prior to sample collection. As shown by Nunes et al. (2020a); Nunes et al., (2020b) and Chen et al. (2020), the number of years under different tillage regimes is linked with changes in soil properties. In our study, conventional tillage could rather be considered as occasional tillage in a no-till system, which had limited effects on crop yields in the meta-study by Peixoto et al. (2020).

In addition to farming practices, genotype was the factor with the greatest effect on yield and N concentrations in wheat. The variety Cellule produced highest yields but with lowest grain N concentrations, whereas Allez-y produced the lowest yields with highest grain N concentrations. This negative correlation between grain yield and grain N concentration has been previously reported (Hawkesford and Riche, 2020). In addition, it is well-known that wheat genotypes differentially absorb and allocate N in their tissues (Swarbreck et al., 2019; Hawkesford and Riche, 2020; Lollato et al., 2021). Wheat breeding takes advantage of this effect to select wheat varieties with a higher N allocation to grains than to other plant tissues as this is better suited for the food industry. Furthermore, breeding aims at finding varieties that produce high yields using less fertilizer to develop more sustainable agriculture (Hirel et al., 2011).

### 4.2 Impact of agricultural management practices on microbial activity

Microbial activity was assessed by analyzing enzymes involved in N and P cycling, namely, protease and phosphatase activity, respectively. The same activities were evaluated by predictive functional analysis based on the 16S rDNA gene sequencing data. Interpretation of enzyme activities subjected to changes in management practices (such as tillage, type of crop, and fertilization) is difficult due to the presence of several direct and indirect effects (Nannipieri et al., 2012). Predictive analysis revealed statistically significant effects on specific enzyme‐encoding genes associated with protease and phosphatase activities, which were influenced by both tillage and fertilization practices. In contrast, these practices as well as genotype had no effects on enzymatic activity. As mentioned above, the trial was newly established on a no-till field site. Thus, agricultural management practices were implemented only a few months prior to sample collection, which could explain why there were no discernible effects on enzymatic activities due to tillage and fertilization. Similarly, Peixoto et al. (2020) have shown that occasional tillage has no effect on microbial activity. Both Chen et al. (2020) and Li et al. (2020) have shown that the number of years under conservation tillage affects microbial diversity directly and indirectly via soil organic C pools, which in turn translates into differences in microbial activity. In general, it can be inferred that no-till practice enhances bacterial activities, attributed to higher organic matter contents and microbial biomass (Nannipieri et al., 2012). Water regime was the only factor affecting microbial activity evaluated by enzymatic assay (**Table 2**). We observed that irrigation increased protease activity compared with rainfed conditions. Also, Brzostek et al. (2012) have shown that protease activity is positively linked with an increase in soil moisture. Mineral N is highly mobile under wet conditions and thus better available for plant uptake but also for microbes, which might have stimulated microbial activity resulting in a higher protease activity. In contrast, rainfed conditions enhanced alkaline phosphatase activity. Also, Borowik and Wyszkowska (2016) and Liu et al. (2019) have shown that alkaline phosphatase activity is enhanced under lower soil moisture conditions. Guangming et al. (2017) reported that phosphatase activity can be positively correlated with soil salinity, which has often been reported to be caused by irrigation (Wang et al., 2015), supporting our observation. In general, effects on enzyme activity can be contradictory as they depend on several factors such as soil, environmental factors, and management practice (Nannipieri et al., 2012). Interpreting the activity of a target enzyme can be challenging as it may be associated with multiple processes.

### 4.3 Effects on microbial community composition and diversity

In this study, we examined changes in microbial community composition at the phyla level. Most abundant bacterial phyla in the rhizosphere and roots of winter wheat were *Actinobacteriota*, *Acidobacteriota*, *Bacteroidota*, *Chloroflexi*, *Gemmatimonadota*, *Myxococcota*, *Planctomycetota*, and *Proteobacteria*. Moreover, the fungal 1 community was dominated by *Ascomycota*, *Basidiomycota*, and *Mortierellomycota*. The microbial phyla described are typical of the rhizosphere and roots of wheat plants grown worldwide, as reported in studies conducted by Azaroual et al. (2022) in Morocco, Azarbad et al. (2022) in Canada, and Wahdan et al. (2023) in Germany.

The second fungal PCR primer set used in this study was expected to be specific for *Glomeromycota*, as described and tested previously (Krüger et al., 2009; Senés-Guerrero et al., 2020). Although the primers were designed to amplify preferentially AMF communities, taxa of other phyla such as *Ascomycota* and *Basidiomycota* were targeted in addition to *Glomeromycota*. Furthermore, around 30% of root-associated OTUs were not assigned to any phyla. It should be noted that DNA sequence databases are still heavily biased toward DNA sequences of *Ascomycota* and *Basidiomycota* and contain less data on *Glomeromycota*, as already noted two decades ago by Hijri et al. (2002). BLAST search of non-assigned sequences revealed that 50% of them were of *Glomeromycota* origin (data not shown), confirming the observation that AMF are still underrepresented. The higher proportion of AMF in the rhizosphere compared with roots may also be attributed to the low coverage and subsequent large number of unassigned sequences in roots. In addition, the presence of an extensive extraradical hyphal network, which represents a significant component of the total AMF biomass, might have added to the higher relative abundance of AMF in rhizosphere (Faghihinia et al., 2023). AMF are well-known for being characteristic of agricultural soils as they form associations with most agricultural crops (Smith and Read, 2010). More diverse cropping systems are known to host more abundant and diverse AMF (Guzman et al., 2021), thanks to their ability to sporulate and to adapt in disturbed environments (Higo et al., 2020). Moreover, we observed a high proportion of *Ascomycota* to colonize wheat roots in addition to AMF. This agrees with the results of Higo et al. (2020), who described them as the most abundant fungal taxa colonizing winter wheat roots.

Analyses of alpha and beta diversity across the entire data set revealed that the habitat was the most discriminating factor shaping microbial communities. Shannon diversity was higher in the rhizosphere compared with roots, and beta-diversity showed a clear compartmentalization between the two habitats for all three microbial communities analyzed. Our results agree with previous studies investigating the impact of plant–soil compartments on microbial community diversity and composition (Bulgarelli et al., 2013; Fitzpatrick et al., 2018; Kuzyakov and Razavi, 2019; Liu et al., 2019). Beckers et al. (2017) observed strong clustering of root- and rhizosphere-associated bacterial communities of field-grown poplar trees. They also found higher diversity and evenness in the rhizosphere compared with roots probably due to plant microenvironments or ecological niches providing different biotic and abiotic gradients and soluble organic compounds (Beckers et al., 2017). Similarly, Coleman-Derr et al. (2016) and Fernández-González et al. (2019) observed higher bacterial and fungal alpha diversity in the rhizosphere compared with roots of agave plants and olive tree, respectively. Studies of Martínez-Diz et al. (2019) on grapevine also found higher fungal diversity in the rhizosphere compared with roots, suggesting that the root tissue entails a barrier for fungal colonization. Also, Maciá-Vicente et al. (2020) described the soil–plant compartment of Brassicaceae as the strongest discriminating factor for fungal communities compared with plant species and sampling time. Driving factors for the observed decline in microbial diversity and richness may be related to two processes controlled by the root surface to filter the entering of soil microbes. Firstly, the attraction of soil microbes with root-colonizing traits via root exudates causes their enrichment in the rhizosphere. Secondly is the ability of selected soil microbes to effectively colonize roots (van der Heijden and Schlaeppi, 2015). This model of microbial recruitment was originally proposed for bacteria, but based on our data, we may assume that the model can also be extended to fungal communities.

### 4.4 Effects of management practices on bacterial and fungal diversity

PERMANOVA analysis revealed the influence of agricultural practices on habitat-specific microbial communities. The interaction of tillage and water regime significantly shaped rhizosphere- and root-associated bacterial and rhizosphere-associated fungal 2 communities. In contrast, rhizosphere- and root-associated fungal 1 communities were impacted by genotype and tillage, respectively. Generally, soil disturbances caused by field management, such as cropping system, tillage, fertilization, and residue management, are among the major factors affecting soil microbial community structure and diversity (Nannipieri et al., 2003; Guo et al., 2016; Tang et al., 2022; Hermans et al., 2023). However, disentangling the effects of agricultural practices on soil microbial community structure is complex. Since synergistic effects of tillage, irrigation, and fertilization on microbial communities have not yet been investigated, the effects are thus first discussed individually. It is well known that tillage practices alter the chemical and physical characteristics of soil such as bulk density, water holding capacity, pore size distribution, and aggregation (Li et al., 2020). However, the effects of tillage on microbial community composition are inconsistent, ranging from tillage-induced changes in bacterial and fungal community composition (Hartman et al., 2018) to no changes (Essel et al., 2019). As Chen et al. (2020) and Li et al. (2020) indicated, soil properties, N input, and duration of conservation tillage are the main factors influencing the extent of tillage effects on microbial communities. However, the effects of occasional or strategic tillage on microbial communities have rarely been studied. In contrast to our results, Rincon-Florez et al. (2020) did not find significant effects of strategic tillage on bacterial and fungal community composition. As the meta-study of Peixoto et al. (2020) showed, occasional tillage can increase microbial biomass, possibly related to greater soil porosity and better access to SOC, which could explain the observed changes in microbial community composition. Irrigation can affect the size, diversity, and structure of soil microbial communities through a broad range of mechanisms including changes in soil nutrient concentration and transport, which locally affects substrate availability as well as SOC (Han et al., 2007; Li et al., 2020). Yuan et al. (2016) also observed that irrigation practice had a stronger effect on the abundance, diversity, and structure of bacterial communities than fertilization, confirming the driving effect of soil moisture on the fluctuation of bacterial communities. The meta-analysis of Xu et al. (2020) helped to evaluate the impact of long-term precipitation changes on microbial communities. They observed that microbial community composition was strongly affected by changes in water availability, showing that drought generally led to a decline in microbial biomass, whereas increased precipitation led to an increase in biomass (Xu et al., 2020), which might further translate into changes in microbial community composition.

To the best of our knowledge, this work was the first to investigate the effects of the interaction between tillage and irrigation on both bacterial and fungal communities. The effects of tillage on soil physical and chemical properties are closely related to the soils’ water holding capacity. Conventional tillage exposes the soil at the surface to wet–dry and freeze–thaw cycles, which increases the turnover of macroaggregates, speeds up the decomposition of soil organic matter, and disrupts the existing pore network (Hartmann and Six, 2023). Furthermore, ploughing leads to the formation of voids between soil aggregates, resulting in reduced infiltration and distribution of water (Hartmann and Six, 2023). In contrast, conservation tillage, as previously mentioned, promotes good soil structure and thus better water storage capacity. Therefore, it is not surprising that we observed an interaction between tillage and irrigation practice on soil microbial communities.

Our findings also revealed that genotype had an impact on rhizosphere-associated fungal 1 community. It is assumed that plant genotypes can alter the composition of the microbiome by secretion of root exudates. Depending on their composition, root exudates can specifically stimulate members of the microbial community, thus actively shaping the structure of rhizosphere microbial communities (Philippot et al., 2013; Busby et al., 2017). However, several studies showed different results regarding the effect of plant genotype. Simonin et al. (2020) found that wheat genotype has a limited effect on rhizosphere bacterial and fungal communities in African and European soils. Latz et al. (2021) observed a genotype effect on root-associated fungal communities in a pot experiment with different wheat genotypes. In any case, our results support the hypothesis that there is a degree of specification in the interaction between crop genotypes and their associated microbiome. We also provide evidence for the potential to exploit specific plant–microbe interactions to incorporate them into plant breeding programs (Peiffer et al., 2013; Philippot et al., 2013; Quambusch et al., 2014; Dubey and Sharma, 2021; Kusstatscher et al., 2021; Gutierrez and Grillo, 2022).

### 4.5 Differentially abundant and prevalent microbial communities in rhizosphere and roots

Differential analysis allows to identify discriminating OTUs being specific for rhizosphere and roots, which was the only distinctive characteristic among all experimental factors investigated. Thus, the mean relative abundance of frequently detected genera in different habitats were compared. Rhizospheric bacteria were significantly enriched in *Blastococcus*, *Skermanella*, and *Entotheonellacea*. Among them, members of *Blastococcus* were reported to be closely linked to soil fertility (Li et al., 2022b). Main bacterial genera in roots were *Lechevalieria*, *Promicromonospora*, *Actinoplanes*, *Streptomyces*, and *Pseudomonas*. These genera belong to *Actinomycetales* or *Pseudomonadales* orders, which are well known to colonize roots of different crops such as tomato and wheat (Aleklett et al., 2022; Zhang et al., 2022). Furthermore, *Streptomyces* and *Pseudomonas* have been described for their plant growth-promoting potential (Romano et al., 2020; Chouyia et al., 2022; Quiroz-Carreño et al., 2022). *Cystofilobasidium*, *Basidioascus*, and *Sporobolomyces* were the most prevalent fungal 1 genera in the rhizosphere; *Sporobolomyces* is one of the major fungal genera commonly identified in the wheat rhizosphere (Levi et al., 2022). Interestingly, *Cystofilobasidium* was reported by Casini et al. (2019) to colonize ancient wheat cultivars but not modern ones, which is considered a possible consequence of plant breeding and domestication on the native microbiome of crops. *Magnaporthiopsis*, the only fungal 1 genus found to be characteristic for wheat roots, has previously been described as pathogenic taxa by Luo et al. (2017). The most frequently detected rhizosphere-associated fungal 2 genera were *Rhizophlyctis* typical of agricultural soils (Gleason et al., 2019), *Nowakowskiella* and the AMF *Scutellospora* (Hijri et al., 2010). *Scutellospora* spp. have also been observed in high abundance in agricultural soils receiving mineral fertilizers by Oehl et al. (2004). In addition to the differentially abundant OTUs, we also examined the most abundant OTUs representing the core community in roots and the rhizosphere. For all investigated communities, root core communities were composed of very few OTUs compared with rhizosphere core communities. Firstly, this might result from the filtering effect of the roots as described earlier and secondly because different wheat genotypes were cultivated. Most of the bacterial and fungal OTUs representing the core community in our study were also detected by Simonin et al. (2020) who described the rhizosphere-associated bacterial and fungal core community of different wheat genotypes. Having a closer look at the fungal 2 core community revealed that most roots were colonized by different Ascomycotan OTUs including *Pleosporales*, *Sordariomycetes*, and *Xylariales* and all of them described as potential plant pathogens and also detected by Simonin et al. (2020). These potential pathogens might have accumulated in the soil with barley as pre-crop and finally infected the wheat roots. Surprisingly, no Glomeromycotan OTU joint the root core community, potentially because AMF are known to be more host specific than pathogenic fungi, and since four winter wheat varieties were cultivated, they might have hosted individual AMF core communities. As expected, the fungal 2 rhizosphere core community was composed of several Glomeromycotan OTUs belonging to the family *Glomeraceae* and *Claroideoglomeraceae*, typically found in agricultural soils (Oehl et al., 2004).

## 5 Conclusions

Our research on four wheat genotypes grown under different agricultural practices resulted in several outcomes: (i) genotype, fertilization, and water regime impacted to different extents wheat yield as well as N allocation in the different plant parts; (ii) water regime affected microbial protease and phosphatase activity; and (iii) the study on bacterial and fungal community structure confirmed that habitat is the most important factor shaping microbial communities. By examining individual habitats, management effects could be studied separately, revealing a strong interacting effect of tillage practices and water regime on rhizosphere and root-associated bacterial and fungal 2 communities, and a sole tillage effect on root-associated fungal 1 communities. Since irrigation and tillage practices have structural effects on the soil, these factors can shape rhizosphere- and root-associated microbial communities. The secretion of specific root exudates by different wheat genotypes has shaped the entire fungal community in the rhizosphere, referred to as fungal 1 community. In such a complex scenario, shifts in microbial communities may indicate possible changes in soil properties.

## Data availability statement

The data presented in the study are deposited in the NCBI Sequence Read Archive (SRA) repository, accession number PRJNA985128.

## Author contributions

AS, NB, FT, HF and SS designed the experiment. GB and MS planned and conducted the field experiment and performed the agronomic analyses, SS, IR, HF, FT, and MD performed the microbial community analyses. IR and NB performed statistical analyses. IR and SS wrote the draft manuscript. All authors revised the final manuscript.
