# Supplementary material for: Impact of conservation tillage on wheat performance and its microbiome
Source: Front Plant Sci. 2023 Aug 21;14:1211758. doi: 10.3389/fpls.2023.1211758 (PMC10475739; doi:10.3389/fpls.2023.1211758)
Supplement: Supplementary file 4 [file DataSheet_1.docx]

Supplementary Material

Impact of conservation tillage on wheat performance and its microbiome

Ida Romano, Natacha Bodenhausen, Gottlieb Basch, Miguel Soares, Hanna Faist, Friederike Trognitz, Angela Sessitsch, Marcé Doubell, Stephan Declerck, Sarah Symanczik*

*** Correspondence:** Sarah Symanczik: [sarah.symanczi@fibl.org](mailto:sarah.symanczi@fibl.org)

# Supplementary Data

Assessing enzyme activity in the rhizosphere

Protease activity was assessed following the protocols described by Schinner (1991), and Ladd and Butler (1972) adapted for 96 well approach. Briefly, 5 g of fresh frozen soil was mixed with 10 ml of Tris-Buffer and vortexed. Then 350 µl of soil slurry was transferred in four technical replicates into a 96 deep well plate, of which three samples were mixed with 250 µl of casein substrate and one sample left as control without casein substrate. Plates were incubated at 50 °C for 180 min and shaken at 130 rpm. After incubation, deep-well plates were immediately put on ice to stop enzyme activity and centrifuged at 5000 x g for 5 min at 10 °C. Then, 250 µl of supernatant was transferred to a clean deep well plate, supplemented with 250 µl TCA (Trichloroacetic acid solution 0.92 M) and only the controls were additionally supplemented with 250 µl of casein substrate. Plates were again centrifuged at 5000 x g for 5 min at 10 °C. Without touching the pellet, 50 μl of supernatant was transferred into a 96-well microplate supplemented with 79 µl of Alkali reagent and 50 µl of the Folin reagent. After 10 min of incubation at room temperature (RT), tyrosine concentration was measured photometrically at 680 nm in a plate reader. Triplicated standard curves were prepared by mixing adequate volumes of Tris-buffer with different concentrations of Tyrosine stock solution (from 0 to 300 µl), 500 µl of Casein stock solution and 500 µl of TCA in 96-well microplates incubated for 10 min at RT and measured at 680 nm. Protease activity was calculated in amount of Tyrosine equivalents, which are released from Sodium-Casein during the incubation time of 3 h according to the following formula:

[(Mean TE in sample – Mean TE in control) *100]/ % TS = TE / (g TS * 3h)

Where TE is Tyrosine equivalents in µg, and TS is the amount of soil dry weight.

Acid and alkaline phosphomonoesterase activity was determined applying the protocols described by Margesin (1993) and Tabatabai and Bremner (1969) adapted for 96 deep-well microplates. Briefly, 5 g of soil was mixed with 10 ml of Modified universal buffer (MUB - pH 6.5 or 11) and vortexed. Then 200 µl of soil slurry was transferred in four technical replicates into a 96 deep well plate, of which three samples were mixed with 50 µl of p-Nitrophenyl Phosphate (pNPP) solution (6.5 pH) and one sample left as blank without pNPP solution. Plates were incubated in a water bath at 37 °C for 60 min. After incubation, 50 µl of CaCl_2_ (0.5 M), 200 µl of NaOH (0.5 M) and 500 µl of demineralized water were directly added to all wells. Then, 50 µl of pNPP (pH 6.5) was added to all blank samples before centrifugation at 5000 x g for5 min. The supernatant was transferred and diluted (10 µl supernatant + 190 µl demineralized water) into microplates, and the absorbance measured at 405 nm. The standard curve was prepared in triplicates by mixing different volumes of nitrophenol standard solution (0 – 22 % of nitrophenol solution), with 10 µl of CaCl_2_ and 40 µl of NaOH adjusted with H_2_O up to a final volume of 1000 µl. Final p-Nitrophenol (µg/g/h) concentration was calculated according to the following formula:

(C x v)/ (EW x t) – (C Bl x v) / (EWBl x t) = p-Nitrophenol (µg/g/h)

Where: C represent µg/ml nitrophenol in filtrate, C Bl is µg/ml nitrophenol in the blank sample, V is the volume of the suspension, EW is the initial soil dry weight (related to dry matter), EWBl is the weight of the blank soil sample (related to dry matter), and t represent the incubation time (1h).

# Supplementary Figures and Tables

## Supplementary Figures

**
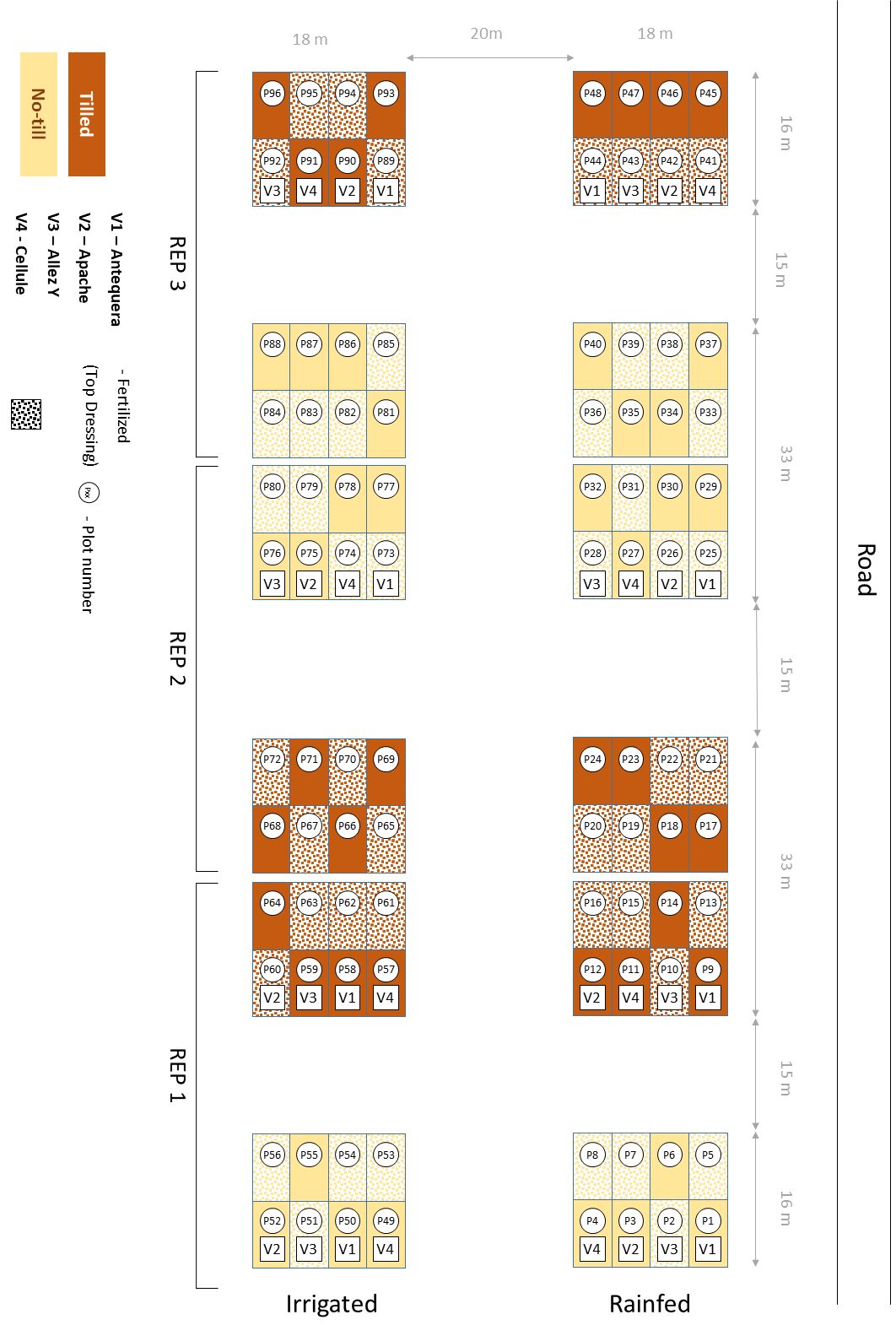
**

**Supplementary Figure 1** Plan of the field trial Beja, Alentejo, Portugal (37° 57' N, -7° 49' E) where four winter wheat genotypes (V1; V1: Antequera, V2: Apache, V3: Allez Y, V4: Cellule) are grown under two tillage practices brown: tilled plots; yellow: no-tilled plots), two fertilization practices (dotted rectangle: full nitrogen fertilization; plain rectangles: half fertilization) and two water regimes (rainfed and irrigated).


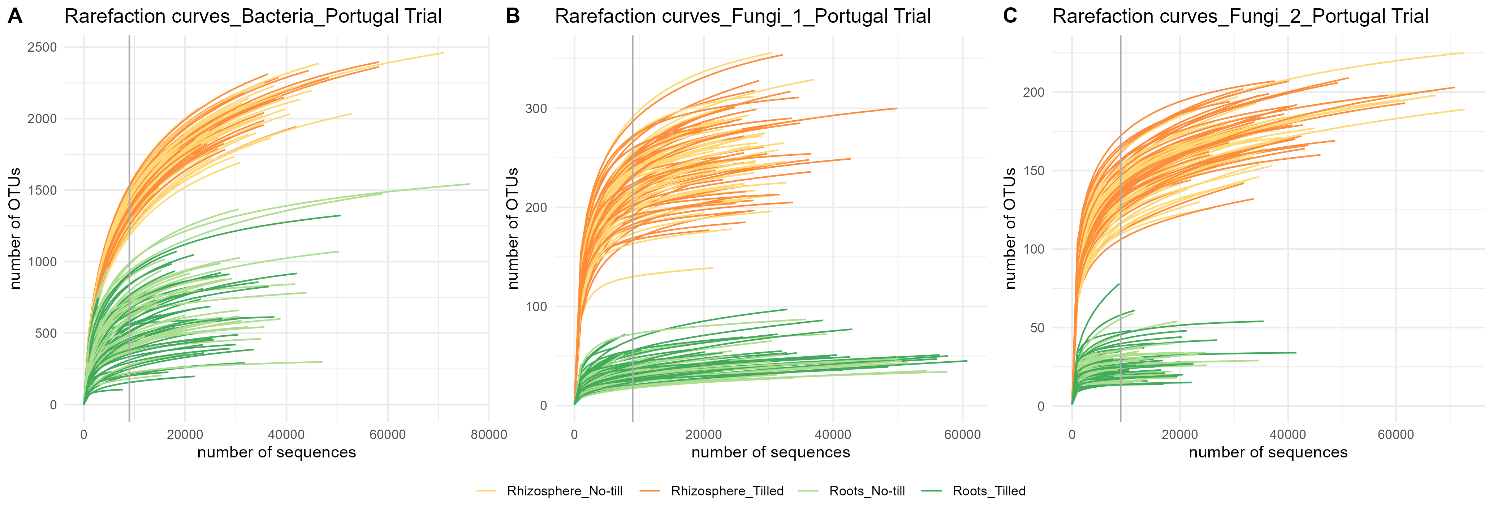


Supplementary Figure 2 Rarefaction curves for bacteria (A), fungi 1 (B), and fungi 2 (C) in the rhizosphere (yellow) and root (green) of winter wheat.


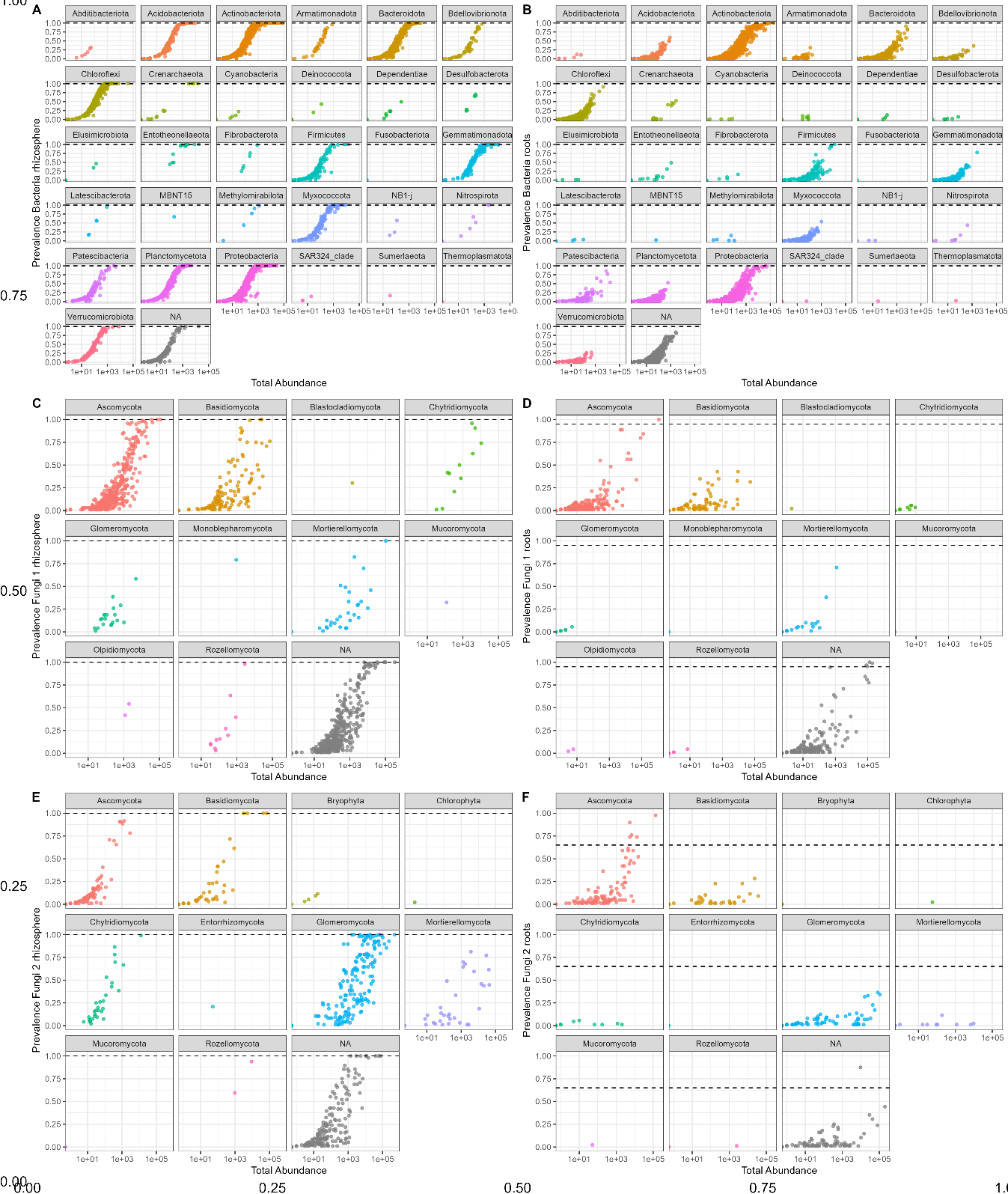


Supplementary Figure 3 Prevalence plots and top OTUs for bacteria (A, B), fungi 1 (C, D) and fungi 2 (E, F) present in the rhizosphere and roots of winter wheat.

**
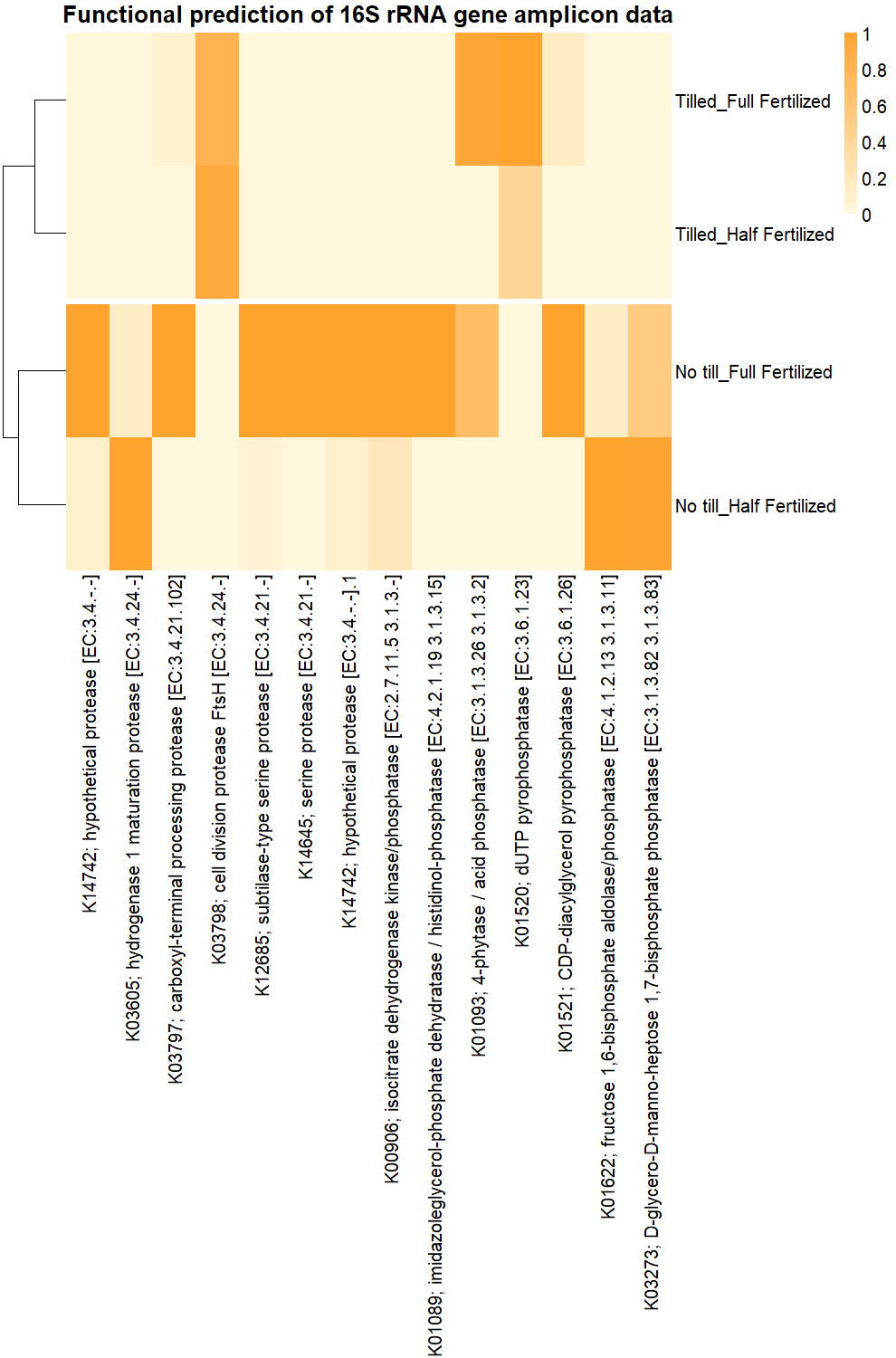
**

**Supplementary Figure 4** Predicted abundances of enzyme-encoding protease and phosphatase genes. The colour code refers to gene abundance, with high predicted abundances (orange) and low predicted abundances (light yellow).

**Supplementary Table 1** Primer sets used for amplification of root- and rhizosphere-associated bacteria and fungi and primer sequences, targeted genes, PCR reaction conditions and references.

| **Microbial community** | **Primer set ^a^** | | **Sequence (5'-3')** | **Targeted gene ^b^** | **PCR reaction conditions ^c^** | | **References** |
| --- | --- | --- | --- | --- | --- | --- | --- |
| Bacteria | F | 799F-*ill* | *TCG TCG GCA GCG TCA GAT GTG TAT AAG AGA CAG AAC* MGG ATT AGA TAC CCK G | V5 and V6 region SSU rRNA gene | Initial denaturation: 95°C for 3 min | | Chelius and Triplett, 2001; Bonder et al., 2012 |
|  |  |  |  |  | Denaturation: 98°C for 30 s | 25 cycles |  |
|  | R | 1175-*ill* | *GTC TCG TGG GCT CGG AGA TGT GTA TAA GAG ACA G* AC GTC RTC CCC DCC TTC CTC |  | Annealing: 55°C for 30 s |  |  |
|  |  |  |  |  | Elongation: 72°C for 30 s |  |  |
|  |  |  |  |  | Final elongation: 72°C for 5 min | |  |
| Fungi 1 | F | ITS1F-*CS1* | *ACACTGACGACATGGTTCTACA* CTT GGT CAT TTA GAG GAA GTA A | ITS1 rRNA gene | Initial denaturation: 95°C for 3 min | | White et al., 1990 |
|  |  |  |  |  | Denaturation: 98°C for 15 s | 35 cycles |  |
|  | R | ITS2-*CS2* | *TACGGTAGCAGAGACTTGGTCT* GCT GCG TTC TTC ATC GAT GC |  | Annealing: 61°C for 30 s |  |  |
|  |  |  |  |  | Elongation: 72°C for 30 s |  |  |
|  |  |  |  |  | Final elongation: 72°C for 5 min | |  |
| Fungi 2 | F | SSUmAf | TGG GTA ATC TTT TGA AAC TTY A  TGG GTA ATC TTR TGA AAC TTC A | LSU rRNA gene | Initial denaturation: 95°C for 3 min | | Krüger et al., 2009; Senés-Guerrero et al., 2020 |
|  |  | LSUD2mod-*ill* | *Ill-*TGG GTA ATC TTR TGA AAC TTC A |  | Denaturation: 98°C for 20 s | 40 cycles |  |
|  | R | LSUmAr | TGG GTA ATC TTT TGA AACTTY A  GCT CTA ACT CAA TTC TAT CGA T  TGC TCT TAC TCA AAT CTA TCA AA  GCT CTT ACT CAA ACC TAT CGA |  | Annealing: 53/*65*°C for 15 s |  |  |
|  |  |  |  |  | Elongation: 72°C for 2 min/ *30 s* |  |  |
|  |  | LSUmBr*-ill* | *Ill-*DAA CAC TCG CAT ATA TGT TAG A  *Ill-* AAC ACT CGC ACA CAT GTT AGA  *Ill-* AAC ACT CGC ATA CAT GTT AGA  *Ill-* AAA CAC TCG CAC ATA TGT TAG A  *Ill-* AAC ACT CGC ATA TAT GCT AGA |  | Final elongation:72°C for 5 min | |  |
| ^a^F: forward primer; R: reverse primer; *ill* is the Illumina-tag with the sequence 5’-TCG TCG GCA GCG TCA GAT GTG TAT AAG AGA CAG-3’ for F-primer and 5’-GTC TCG TGG GCT CGG AGA TGT GTA TAA GAG ACA G-3’ for R-primer. | | | | | | | |
| ^b^SSU rRNA: small subunit ribosomal RNA; ITS: internal transcribed spacer; LSU: large subunit ribosomal RNA | | | | | | | |
| ^c^For characterizing the fungi 2 community, the amplification of the targeted gene was performed using a nested PCR with the primer mixtures SSUmAf and LSUmAr as the primary PCR. The nested PCR was performed using ten-time diluted products of the first PCR reaction amplified by LSUD2mod/LSUmBr primer mix. PCR reaction conditions were almost equal for both primer sets, except for differences in annealing temperature and elongation time of the nested PCR as shown in italic. | | | | | | | |

**Supplementary Table 2** Yield and nitrogen (N) concentrations in spikes, leaves and stalk, grains, and total N uptake of four winter wheat genotypes grown under different water and fertilization regimes and tillage practices. Values represent means ± standard error of three replicates.

See Excelfile “Supplementary Table 2”

**Supplementary Table 3** Analysis of Variance of Shannon Index for bacteria (A), fungi 1 (B), and fungi 2 (C) in the rhizosphere and root of winter wheat.

|  | **Df Sq** | **Sum Sq** | **Mean Sq** | **F value** | **Pr(>F)** |  |
| --- | --- | --- | --- | --- | --- | --- |
| 1. **Bacteria** | | | | | | |
| **Habitat** | 1 | 8117033 | 8117033 | 1277.413 | <2e-16 | *** |
| **Water regime** | 1 | 253 | 253 | 0.040 | 0.8420 |  |
| **Tillage** | 1 | 1866 | 1866 | 0.294 | 0.5886 |  |
| **Fertilization** | 1 | 2709 | 2709 | 0.426 | 0.5147 |  |
| **Genotype** | 3 | 1673 | 558 | 0.088 | 0.9667 |  |
| 1. **Fungi 1** | | | | | | |
| **Habitat** | 1 | 65950 | 65950 | 1013.967 | <2e-16 | *** |
| **Water regime** | 1 | 52 | 52 | 0.802 | 0.372 |  |
| **Tillage** | 1 | 1 | 1 | 0.022 | 0.881 |  |
| **Fertilization** | 1 | 55 | 55 | 0.859 | 0.357 |  |
| **Genotype** | 3 | 514 | 171 | 2.63 | 0.051 | . |
| 1. **Fungi 2** | | | | | | |
| **Habitat** | 1 | 34510 | 34510 | 1253.387 | <2e-16 | *** |
| **Water regime** | 1 | 0 | 0 | 0.010 | 0.921 |  |
| **Tillage** | 1 | 23 | 23 | 0.825 | 0.365 |  |
| **Fertilization** | 1 | 11 | 11 | 0.414 | 0.521 |  |
| **Genotype** | 3 | 11 | 4 | 0.128 | 0.943 |  |
| *Signif. codes: 0 ’***’ 0.001 ’**’ 0.01 ’*’ 0.05 ’.’ 0.1 ’ ’ 1* | | | | | | |

**Supplementary Table 4** Output of PERMANOVA analysis for bacteria, fungi 1, and fungi 2 in the rhizosphere and root of winter wheat analyzed jointly, and separately.

|  | **Factor** | **Df** | **SumsOfSqs** | **MeanSqs** | **F model** | **R2** | **Pr(>F)** |  |
| --- | --- | --- | --- | --- | --- | --- | --- | --- |
| **Bacteria** |  |  |  |  |  |  |  |  |
| **Permutation free** | **Habitat** | 1 | 16.263 | 16.263 | 109.070 | 0.385 | 0.001 | *** |
|  | **Water regimes** | 1 | 0.304 | 0.304 | 2.036 | 0.007 | 0.067 | . |
|  | **Tillage** | 1 | 0.202 | 0.202 | 1.355 | 0.005 | 0.172 |  |
|  | **Fertilization** | 1 | 0.104 | 0.104 | 0.698 | 0.003 | 0.627 |  |
|  | **Genotype** | 3 | 0.491 | 0.16 | 1.098 | 0.012 | 0.310 |  |
| **Root** | **Water regimes** | 1 | 1.147 | 1.147 | 4.006 | 0.041 | 0.001 | *** |
|  | **Tillage** | 1 | 0.459 | 0.459 | 1.602 | 0.017 | 0.055 | . |
|  | **Fertilization** | 1 | 0.206 | 0.206 | 0.721 | 0.007 | 0.846 |  |
|  | **Genotype** | 3 | 0.80 | 0.269 | 0.939 | 0.029 | 0.568 |  |
| **Rhizosphere** | **Water regimes** | 1 | 0.113 | 0.113 | 2.657 | 0.028 | 0.003 | ** |
|  | **Tillage** | 1 | 0.075 | 0.076 | 1.776 | 0.018 | 0.029 | * |
|  | **Fertilization** | 1 | 0.059 | 0.052 | 1.224 | 0.013 | 0.175 |  |
|  | **Genotype** | 3 | 0.103 | 0.034 | 0.810 | 0.025 | 0.869 |  |
| **Fungi 1** |  |  |  |  |  |  |  |  |
| **Permutation free** | **Habitat** | 1 | 20.748 | 20.748 | 93.323 | 0.335 | 0.001 | *** |
|  | **Water regimes** | 1 | 0.282 | 0.282 | 1.268 | 0.005 | 0.218 |  |
|  | **Tillage** | 1 | 0.349 | 0.349 | 1.568 | 0.006 | 0.131 |  |
|  | **Fertilization** | 1 | 0.155 | 0.155 | 0.696 | 0.003 | 0.687 |  |
|  | **Genotype** | 3 | 1.003 | 0.334 | 1.504 | 0.016 | 0.063 | . |
| **Root** | **Water regimes** | 1 | 0.346 | 0.346 | 1.35341 | 0.01518 | 0.191 |  |
|  | **Tillage** | 1 | 0.537 | 0.537 | 2.10160 | 0.02357 | 0.046 | * |
|  | **Fertilization** | 1 | 0.121 | 0.121 | 0.47303 | 0.00530 | 0.921 |  |
|  | **Genotype** | 3 | 0.829 | 0.276 | 1.08189 | 0.03640 | 0.324 |  |
| **Rhizosphere** | **Water regimes** | 1 | 0.208 | 0.208 | 0.923 | 0.011 | 0.275 |  |
|  | **Tillage** | 1 | 0.161 | 0.161 | 0.843 | 0.009 | 0.757 |  |
|  | **Fertilization** | 1 | 0.133 | 0.133 | 0.699 | 0.007 | 0.947 |  |
|  | **Genotype** | 3 | 0.899 | 0.300 | 1.573 | 0.049 | 0.004 | ** |
| **Fungi 2** |  |  |  |  |  |  |  |  |
| **Permutation free** | **Habitat** | 1 | 20.257 | 20.257 | 81.983 | 0.308 | 0.001 | *** |
|  | **Water regimes** | 1 | 0.465 | 0.4653 | 1.883 | 0.007 | 0.075 | . |
|  | **Tillage** | 1 | 0.555 | 0.555 | 2.248 | 0.009 | 0.026 | * |
|  | **Fertilization** | 1 | 0.233 | 0.233 | 0.942 | 0.004 | 0.401 |  |
|  | **Genotype** | 3 | 0.758 | 0.253 | 1.023 | 0.012 | 0.388 |  |
| **Root** | **Water regimes** | 1 | 0.493 | 0.493 | 1.363 | 0.016 | 0.139 |  |
|  | **Tillage** | 1 | 0.373 | 0.373 | 1.030 | 0.012 | 0.395 |  |
|  | **Fertilization** | 1 | 0.285 | 0.285 | 0.767 | 0.009 | 0.689 |  |
|  | **Genotype** | 3 | 1.095 | 0.365 | 1.008 | 0.035 | 0.484 |  |
| **Rhizosphere** | **Water regimes** | 1 | 0.370 | 0.370 | 2.364 | 0.027 | 0.002 | ** |
|  | **Tillage** | 1 | 0.507 | 0.507 | 3.619 | 0.036 | 0.001 | *** |
|  | **Fertilization** | 1 | 0.183 | 0.183. | 1.305 | 0.013 | 0.152 |  |
|  | **Genotype** | 3 | 0.407 | 0.135 | 0.969 | 0.029 | 0.539 |  |
| *Signif. codes: 0 ’***’ 0.001 ’**’ 0.01 ’*’ 0.05 ’.’ 0.1 ’ ’ 1* | | | | | | | | |

**Supplementary Table 5** Microbial core communities (prevalent OTUs) in roots and the rhizosphere of winter wheat.

See “Supplementary Table 5”

**Supplementary Table 6** Results of the analysis of variance (ANOVA) conducted to assess the variations in predicted enzyme-encoding functional genes of rhizosphere of four winter wheat genotypes grown under different water and fertilization regimes and tillage practices. The dataset used for the analysis consisted of proteases and phosphatase enzyme-encoding genes predictions obtained through Tax4fun R package.

Excelfile “Supplementary Table 6”

# References

Bonder, M.J., Abeln, S., Zaura, E., and Brandt, B.W. (2012) Comparing clustering and pre-processing in taxonomy analysis. Bioinformatics 28: 2891–2897.

Chelius, M.K., and Triplett, E.W. (2001) The diversity of Archaea and bacteria in association with the roots of Zea mays L. Microb Ecol 41: 252–263.

Krüger, M., Stockinger, H., Krüger, C., and Schüßler, A. (2009). DNA‐based species level detection of Glomeromycota: one PCR primer set for all arbuscular mycorrhizal fungi. New Phytol. 183, 212–223. doi: 10.1111/j.1469-8137.2009.02835.x.

Ladd, J. N., and Butler, J. H. A. (1972). Short-term assays of soil proteolytic enzyme activities using proteins and dipeptide derivatives as substrates. Soil Biol. Biochem. 4, 19–30. doi: 10.1016/0038-0717(72)90038-7.

Margesin, R. (1993). “Bestimmung der sauren und alkalischen Phosphomonoesterase-Aktivität,” in Bodenbiologische Arbeitsmethoden (Springer Berlin, Heidelberg, New York), 200–203.

Schinner, F. . R. Ö. & E. K. (1991). Bodenbiologische Arbeitsmethoden. Springer, Berlin 1991. 230 Seiten, 4 Abb., DM 58, -. ISBN 3-540-53143-2. Zeitschrift für Pflanzenernährung und Bodenkd. 154, 315. doi: 10.1002/jpln.19911540414.

Senés-Guerrero, C., Giménez, S., Pacheco, A., Gradilla-Hernández, M. S., and Schüßler, A. (2020). New MiSeq based strategy exposed plant-preferential arbuscular mycorrhizal fungal communities in arid soils of Mexico. Symbiosis 81, 235–246. doi: 10.1007/s13199-020-00698-5

Tabatabai, M. A., and Bremner, J. M. (1969). Use of p-nitrophenyl phosphate for assay of soil phosphatase activity. Soil Biol. Biochem. 1, 301–307.

White, T. J., Bruns, T., Lee, S. J. W. T., & Taylor, J. (1990). Amplification and direct sequencing of fungal ribosomal RNA genes for phylogenetics. PCR protocols: a guide to methods and applications, 18(1), 315-322.
